# Patient-specific modeling of individual sickle cell behavior under transient hypoxia

**DOI:** 10.1371/journal.pcbi.1005426

**Published:** 2017-03-13

**Authors:** Xuejin Li, E. Du, Ming Dao, Subra Suresh, George Em Karniadakis

**Affiliations:** 1 Division of Applied Mathematics, Brown University, Providence, Rhode Island, United States of America; 2 Department of Materials Science and Engineering, Massachusetts Institute of Technology, Cambridge, Massachusetts, United States of America; 3 Department of Ocean and Mechanical Engineering, Florida Atlantic University, Boca Raton, Florida, United States of America; 4 Department of Biomedical Engineering, Computational Biology Department, and Department of Materials Science and Engineering, Carnegie Mellon University, Pittsburgh, Pennsylvania, United States of America; University of California San Diego, UNITED STATES

## Abstract

Sickle cell disease (SCD) is a highly complex genetic blood disorder in which red blood cells (RBC) exhibit heterogeneous morphology changes and decreased deformability. We employ a kinetic model for cell morphological sickling that invokes parameters derived from patient-specific data. This model is used to investigate the dynamics of individual sickle cells in a capillary-like microenvironment in order to address various mechanisms associated with SCD. We show that all RBCs, both hypoxia-unaffected and hypoxia-affected ones, regularly pass through microgates under oxygenated state. However, the hypoxia-affected cells undergo sickling which significantly alters cell dynamics. In particular, the dense and rigid sickle RBCs are obstructed thereby clogging blood flow while the less dense and deformable ones are capable of circumnavigating dead (trapped) cells ahead of them by choosing a serpentine path. Informed by recent experiments involving microfluidics that provide *in vitro* quantitative information on cell dynamics under transient hypoxia conditions, we have performed detailed computational simulations of alterations to cell behavior in response to morphological changes and membrane stiffening. Our model reveals that SCD exhibits substantial heterogeneity even within a particular density-fractionated subpopulation. These findings provide unique insights into how individual sickle cells move through capillaries under transient hypoxic conditions, and offer novel possibilities for designing effective therapeutic interventions for SCD.

## Author summary

Sickle cell disease is a genetic blood disease that causes vaso-occlusive pain crises. Here, we investigate the individual sickle cell behavior under controlled hypoxic conditions through patient-specific predictive computational simulations that are informed by companion microfluidic experiments. We identify the different dynamic behavior between individual sickle RBCs and normal ones in microfluidic flow, and analyze the hypoxia-induced alteration in individual cell behavior and single-cell capillary obstruction under physiological conditions.

## Introduction

In research investigations of hematological disorders, most experiments are performed on groups of cells with the underlying assumption that all of the cells in a particular *type* are identical. However, recent evidence reveals that individual cells within the same population may differ drastically in size, shape, mechanical properties and protein levels, and these variations can have important consequences for the health and biological function of the entire cell population [1]. A representative case is sickle cell disease (SCD), one of the most common inherited genetic blood disorders affecting more than 270,000 new patients each year [2, 3].

SCD has been characterized as the first molecular disease [4], being linked to the mutation of a single nucleotide in the hemoglobin molecule. The primary pathophysiological event in SCD is the polymerization of sickle hemoglobin (HbS) into long fibers upon deoxygenation (DeOxy) [5, 6]. The fibers distort RBCs into irregular and heterogeneous shapes—e.g. granular, elongated, oval, and crescent (classic sickle) shapes [7, 8]. The hypoxia-affected RBCs are also heterogeneous in their cell density in a range of less than 30 g/dL to more than 46 g/dL [9], which are usually fractioned into four arbitrary cell density subpopulations (fractions I-IV) *in vitro* analysis [7]. Heterogeneous cell fractions engender heterogeneity in cell rigidity [10–13]. These hypoxia-affected RBCs are more sticky and stiff, causing frequent painful episodes of vaso-occlusion and depriving oxygen from tissues and organs [10,14]. The decrease in RBC deformability contributes to impaired blood flow and other pathophysiological origins of the disease. However, the clinical expression of SCD is heterogeneous, as the hypoxia-affected RBCs do not all behave in the same way all the time, and the variance is considerable even within a same density-fraction, making it hard to predict the risk of a vaso-occlusive crisis [15–17]. This poses a serious challenge for disease management.

Precision medicine [18], which accounts for individual variability, is an emerging approach for treatment and prevention of disease [19,20]. Developing such an approach, however, inevitably requires resolving various heterogeneity-related issues, both at whole cell population and single-cell levels [21]. Although developments in quantitative, *in vitro* microfluidic assays provide greater understanding of cell dynamics under hypoxic conditions in patients’ blood samples [16], there is a critical need to develop single-cell level assays to assess sickle RBC behavior under transient hypoxic conditions for better therapeutic interventions [22]. These considerations lead to the motivation for the present work whose aim is to address the following question: To what extent does morphological sickling affect cell dynamics in microcapillaries under transient hypoxia, with consequences for hemodynamics and vaso-occlusion?

In order to gain a better understanding of vaso-occlusive crisis, it is necessary to obtain direct and *real-time* observations of the traversal individual sickle RBCs through microcapillaries and of alterations in cell biodynamics and biorheology in response to controlled changes in oxygen (O_2_) concentration. However, owing to the phenotypic heterogeneity of SCD, existing experimental capabilities do not readily provide this information. As a result, there is a critical need to develop predictive, patient-specific cell models to quantify alterations in cell biodynamics under transient hypoxic conditions. Such models could be validated by recourse to a variety of well-controlled and independent *in vitro* experiments.

In this article, we investigate the dynamic behavior of individual sickle cells in real time, in a capillary-like microenvironment, and perform quantitative analysis of hypoxia-induced alteration in cell behavior and response to obstruction of capillaries by combining predictive simulations with controlled and quantitative information obtained from microfluidic experiments.

## Materials and methods

### Sickle RBC samples

De-identified SCD blood samples from two patients with HU therapy (on-HU) and two patients without HU therapy (off-HU) were selected for this study, following institutional review board (IRB) approvals from the National Institutes of Health (NIH) and Massachusetts Institute of Technology (MIT). All samples were collected into EDTA anticoagulant and stored at 4°C during shipping and storage. Table 1 shows selected hematologic and hemorheologic parameters in these four blood samples. The experiments were performed exactly in the same way as those in Ref. [23], but with a specific focus on (1) correlations between cell shape, mean corpuscular hemoglobin concentration (MCHC), transit velocity and trajectories through the microgates, and (2) movements of individual cells with and without disturbances from adjacent blockages.

**Table 1:** Selected hematologic parameters from four SCD patients.

	Off-HU		On-HU	
	S-P-I	S-P-II	S-P-III	S-P-IV
**Hct, %**	22.9	18.6	21.9	29.2
**MCV, fL**	83.0	83.3	99.1	99.0
**MCHC, g/dL**	36.7	36.6	35.6	34.2
**HbS, %**	84.2	90.1	72.4	86.0
**HbF, %**	11.9	6.0	24.1	10.0
**HbA, %**	0.0	0.0	0.0	0.0
**HbA**_**2**_**, %**	3.9	3.9	3.5	4.0

The symbols S-P-I and S-P-II represent two blood samples from SCD patients not treated with HU, whereas S-P-III and S-P-IV represent the other two blood samples from SCD patients treated with HU. Hct, hematocrit; MCV, mean corpuscular volume; MCHC, mean corpuscular hemoglobin concentration; HbA, hemoglobin A (α_2_β_2_), also known as adult hemoglobin; HbA_2_, a normal variant of hemoglobin A (α_2_δ_2_); HbF, fetal hemoglobin (α_2_γ_2_); HbS, sickle hemoglobin (α_2_β^S^_2_).

After washing twice with PBS (Thermo Scientific) at 821 × *g* for 5 min at 21°C, the RBCs in the blood sample were separated into four density fractions, using a stepwise gradient medium prepared with OptiPrep medium (Sigma Aldrich, Saint Louis, MO, USA) and Dulbecco’s PBS (HyClone Laboratories, Inc., South Logan, UT, USA). The estimated MCHC values were 27.3, 30.9, 34.9, and > 45.0 g/dL for fraction I-IV, respectively (Table 2). Fraction I (SS1, reticulocyte rich) and fraction II (SS2, discocyte rich) have moderate MCHC values, which are similar to those of healthy RBCs. Fractions III (SS3) and IV (SS4) mainly comprised rigid discocytes and irreversible sickle cells (ISCs), respectively, with their MCHC values considerably higher than those of healthy RBCs. The fractionated RBCs were washed with PBS and re-suspended in RPMI-1640 medium with 1% (wt/vol) Bovine Serum Albumin (Sigma-Aldrich, Saint Louis, MO, USA) for cell sickling measurement in a polydimethylsiloxane (PDMS)-based microfluidic hypoxia assay [23]. This platform provided measurements of cell sickling under controlled O_2_ concentrations at 37°C, including a fully Oxy state (20 vol% O_2_), and a hypoxic condition, in which the O_2_ concentration decreased from 20 vol% to below 5 vol% within 15 s and maintained at 2 vol% for the rest periods. Patient-specific data relevant to the present work along with cell morphological data are provided in Table A in S1 Text for predictive simulations.

**Table 2:** Values of cell density and MCHC in different cell fractions.

Cell fraction	I	II	III	IV
**Cell density, g/ml**	1.081	1.091	1.100	1.111
**MCHC, g/dL**	27.3	30.9	34.9	> 45.0

### Computational model and method

**Fig 1:**
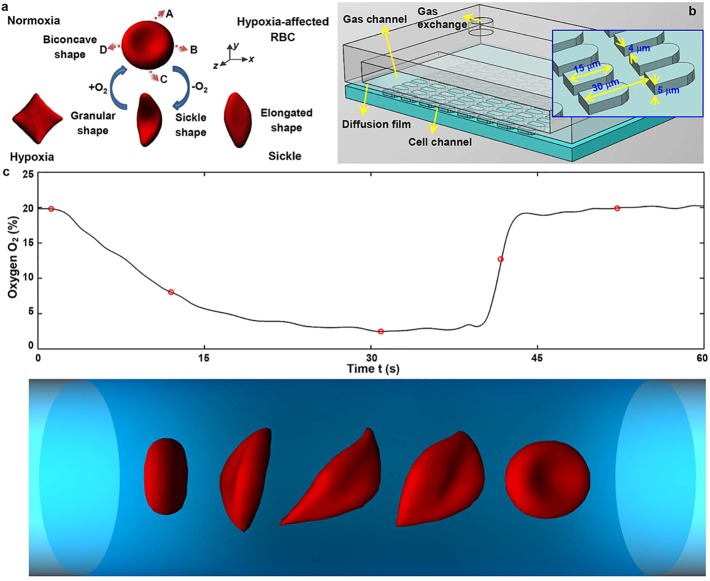
Time-dependent cell morphological sickling model. (a) Morphological transition of RBCs from the biconcave to different sickle shapes under transient hypoxic conditions. Labels **A**–**D** represent the position of the anchor points, which are represented by *εN*_*v*_ (*ε* = 0.016) vertices. (b) Schematic of microfluidic device with O_2_ control for kinetics of cell morphological sickling and unsickling as well as single cell biorheology test. The microfluidic channel contains periodic obstacles forming 15-μm-long, 4-μm-wide and 5-μm-high microgates, mimicking the size of the smallest capillaries in human body. (c) Cell sickling and unsickling process in response to change in O_2_ concentration as a function of time (from companion experiments). Simulations of five sequential snapshots of the RBC shape evolution at O_2_% = 19.8%, 8.1%, 2.5%, 12.7%, and 19.9%, from left to right. These conditions correspond to the red circles indicated in the O_2_ versus time plot. See S1 Video for dynamic shape changes occurring in response to alterations in oxygen concentration.

In order to investigate the behavior of sickle RBCs in each density-fractionated subpopulation under transient hypoxic conditions, we have developed a unified modeling framework based on dissipative particle dynamics (DPD). Our computational framework is predicated on recent developments [23] in microfluidics that quantify *in vitro* single RBC dynamic response and its heterogeneity under controlled oxygen partial pressures in blood samples from SCD patients. Specifically, the geometry of microfluidic channel (Fig 1) and profile of transient hypoxia (Fig 1 and Figure A in S1 Text) are the same as in the experimental setup [23]. The solid walls of the microfluidic channel in DPD simulations are modeled by layers of frozen DPD particles, and a force-adaptive is employed for fluid particles to control their density fluctuations [24]. An external body force is exerted on each fluid particle to generate a flow in the microfluidic channel. In this study, the externally applied body force works in the direction of flow (*x*-direction) on the fluid particles.

We studied the dynamics of sickle RBCs under transient hypoxia using a multiscale RBC model [25]. For completeness, the model is briefly summarized below, whereas details of this MS-RBC model are available in Refs. [25,26]. Here, the membrane of MS-RBC is modeled by a two-dimensional triangulated network with *N*_*v*_ vertices, which are connected by *N*_*s*_ viscoelastic bonds to impose proper membrane mechanics. In this study, the elastic energy of the bond is taken as

$$V_{s}=\sum_{j\in 1\ldots ,N_{s}}\left[\frac{k_{\text{B}}Tl_{m}(3x_{j}^{2}-2x_{j}^{3})}{4p(1-x_{j})}+\frac{k_{p}}{(n-1)l_{j}^{n-1}}\right],$$ (1)

where *l*_*j*_ and *l*_*m*_ are the equilibrium (initial edge) length and maximum extension of spring *j*, *p* is the persistence length, *k*_*p*_ is a constant factor of the spring, and *n* is an exponent. The bending energy of the RBC membrane is written as

$$V_{b}=\sum_{j\in 1\ldots ,N_{s}}k_{b}\left[1-\cos(\theta_{j}-\theta_{0})\right],$$ (2)

where *k*_*b*_ is the bending constant, *θ*_*j*_ and *θ*_0_ are the instantaneous and spontaneous angles between two adjacent triangles with a shared common edge *j*. In addition, the volume and surface area of RBC are controlled to mimic the incompressible interior fluid and the area-preserving cell membrane. The corresponding energy is taken as

$$V_{a+v}=\sum_{j\in 1\ldots ,N_{t}}\frac{k_{d}{\left(A_{j}-A_{0}\right)}^{2}}{2A_{0}}+\frac{k_{a}{\left(A-A_{0}^{\text{tot}}\right)}^{2}}{2A_{0}^{\text{tot}}}+\frac{k_{v}{\left(V-V_{0}^{\text{tot}}\right)}^{2}}{2V_{0}^{\text{tot}}},$$ (3)

where *k*_*a*_ and *k*_*d*_ are the global and local area constraint coefficients, *k*_*v*_ is the volume constraint coefficients, *A*_0_ is the triangle area, and the terms *A*_0_^tot^ and *V*_0_^tot^ are the total area and volume in equilibrium, respectively.

The RBC membrane interacts with the fluid and wall particles through DPD forces, and the system temperature is maintained by the DPD thermostat. The surrounding external fluids and cytosol are modeled by collections of free coarse-grained particles and their separation is enforced through bounce-back reflections at the moving surface of RBC membrane. In addition, we consider a positive correlation between the number of intracellular coarse-grained particles and the MCHC value, i.e., for a denser RBC with a higher MCHC value, we include more coarse-grained particles inside the cell. The MS-RBC model has been validated by comparisons with a number of available experiments that examine the mechanics and biorheology of healthy and diseased RBCs [25–31]. Such connections between simulations and experiments have also indicated that the results developed here are not dependent on the details of refinement of the simulation in that they are independent of the level of coarse-graining for *N*_*v*_ ≥ 500 [25,26]. In this study, we employ a MS-RBC model with a level of coarse-graining (*N*_*v*_ = 500) that facilitates computationally efficient simulations of RBCs in microfluidic channel.

Sickle RBCs undergo various morphological transitions, due to the polymerization of intracellular HbS molecules, from the normal biconcave shape to an irregular sickled shape, in the form of granular, elongated and crescent shapes. Instead of directly modeling the formation of HbS polymer fibers and the resulting cell morphology, we consider an “effective surface tension” (stretching force) applied on the cell membrane to mimic the cell distortion exerted by the growing HbS polymer domain [31,32]. We develop a two-step RBC morphological sickling model, as described below.

1) We first apply surface tension on the cell membrane to transform the discocyte RBC into a granular, elongated or crescent shape. Here, we define the directions along the two long axes of RBC as *x*- and y-directions, and the third direction, perpendicular to the long axes, as the *z*-direction. We choose four anchor points located at the maximum/minimum values in the *x*/*y* directions (Fig 1), where the intracellular polymer fibers can potentially interact with the red cell membrane. Different combinations of the stretching force applied on the anchor points cause different sickle cell shapes: when the stretching force is exerted on two diametrically opposite anchor points (for example, points A and C), an elongated or a crescent cell shape is obtained. Similarly, if the stretching force is exerted on all the four anchor points, a granular shape results. The values of stretching forces for obtaining different shapes of sickle RBCs are shown in Table B in S1 Text. We define the distorted shape as the equilibrium state of the sickle RBC with minimum free energy. Specifically, we adjust the equilibrium length, *l*_*j*_, of each spring *j* to the edge length, *l*_*j*,REF_, of the final distorted state to eliminate local stress on the cell membrane.

2) Subsequently, we employ the kinetic description for sickling RBCs [33] to model the cell morphological sickling process under transient hypoxia. Here, we define the two parameters, *l*_*j*_ and *l*_*j*,REF_, as the reference length of the spring *j*, respectively, in the RBC under normal Oxy state (~20 vol% O_2_) and fully DeOxy state (~2 vol% O_2_). Once the oxygen partial pressure, *p*_O2_, is lower than a critical value *p*_O2,c_ for cell sickling, we assume that a sufficiently large stretching force, *f*_*j*_(*t*), is applied on each spring so that its instantaneous edge length, *l*_*j*_*(t)*, is essentially equal to the expected edge length,

$$l_{j,\text{ref}}(t)=l_{j}\left\{1-\left[\frac{l_{j,}{}_{\text{REF}}}{l_{j}}-1\right]\left[\frac{p_{{\text{O}}_{2}}{}_{,\text{c}}-p_{{\text{O}}_{2}}\left(t-t_{D}\right)}{p_{{\text{O}}_{2}}{}_{,\text{c}}}\right]\right\},$$ (4)

where *t*_D_ is delay time for cell sickling. In the present study, the parameters of *p*_O2,c_, *p*_O2_(*t*), and *t*_D_ are directly obtained or calculated from experiments [23]. Through this approach, we are able to dynamically adjust the bond lengths of the elastic springs to affect the sickling and unsickling processes of RBCs while maintaining the cell membrane free of any total external force.

Membrane stiffening of sickle RBCs is closely related to their morphologic change [11]. It seems likely that a step increase in rigidity occurs at the same time as morphological sickling. Similarly, the shear modulus, *μ*_ref_(*t*), of the hypoxia-affected cell membrane is taken as

$$\mu_{\text{ref}}(t)=\mu_{0}\left\{1-\left[\frac{\mu_{\text{REF}}}{\mu_{0}}-1\right]\left[\frac{p_{{\text{O}}_{2}}{}_{,\text{c}}-p_{{\text{O}}_{2}}\left(t-t_{D}\right)}{p_{{\text{O}}_{2}}{}_{,\text{c}}}\right]\right\},$$ (5)

where *μ*_REF_ is the shear modulus of sickle RBC, described below. The cell unsickling process is analogous to the cell sickling process, by considering the delay time (*t*_D,R_) of cell unsickling. This kinetic cell sickling model, which accounts for (1) cell morphological change, (2) cell sickling and unsickling delay times, and (3) cell membrane stiffening under transient hypoxia, offers an effective method of investigating the sickling and unsickling processes of RBCs in response to changes in O_2_ concentration under both static and flow conditions (S1 Video).

In this study, we modeled a healthy RBC using the multiscale RBC model with the following parameters: number of RBC vertices *N*_*v*_ = 500; RBC area *A*_0_ = 135.2 μm^2^ and volume *V*_0_ = 92.4 μm^3^; RBC membrane bending modulus *k*_*c*,0_ = 2.4 × 10^−19^ J; shear modulus *μ*_0_ = 4.7 pN∙μm^-1^; cytosol viscosity *η*_0_ = 1.2 cp. In contrast to a healthy RBC, the SCD RBC is characterized by extensive impairment in deformability, the extent of which is dependent on the cell density and oxygen partial pressure. Sickle RBCs in different cell density subpopulations exhibit different membrane elasticity in the Oxy state (O_2_ concentration ~ 20 vol%) and the DeOxy state (O_2_ concentration < 5 vol%). Previous studies have shown that the intracellular polymerization of HbS upon DeOxy leads to significant increases in cytosol viscosity and membrane elasticity [34–39]. The cytosol viscosity, *η*_cytosol_, under DeOxy state could be two orders of magnitude greater than *η*_0_ [34,39]. In our previous sensitivity study, we have shown that the blood dynamics is nearly independent of cytosol viscosity if *η*_cytosol_ > 50*η*_0_ [32]. We would expect that the cytosol viscosity also plays a major role in determining sickle cell behavior in a capillary-like microenvironment under transient hypoxia. However, we find the cytosol viscosity in the densest cell fractions IV is only around 4.5 *η*_0_, because our model does not explicitly include the intracellular HbS polymer fibers. Here, we used an effective shear modulus of RBCs, which accounts for the effects of (1) membrane stiffening of sickle RBCs, (2) high membrane tension induced by the growth of intracellular HbS polymers, and (3) elevated cytosol viscosity. In the Oxy state, we select experimentally determined shear modulus data [35–37], and set the effective shear modulus to *μ*_REF_ = 1.0*μ*_0_, 1.2*μ*_0_, 1.5*μ*_0_ and 3.0*μ*_0_ for RBCs in fractions I, II, III and IV, respectively. In the DeOxy state, we consider four distinct types of sickle RBCs with different cell membrane mechanical properties based on experimental observations [11,40]. For an SS1 deformable cell with a low MCHC value, the measured shear modulus increased by up to 10 times after the occurrence of sickling [11], and hence we set the effective shear modulus *μ*_REF_ = (5–10) *μ*_0_. For an SS2 cell with a mild MCHC value, in which the effective shear modulus is increased by one or two orders of magnitude compared to that of healthy RBCs [11,40], we set *μ*_REF_ = (50–100) *μ*_0_. For an ISC of the SS4 type, its effective shear modulus is at least two or three orders of magnitude greater than the value of healthy RBCs [11], so we set *μ*_REF_ = (1000–2000) *μ*_0_. For a SS3 rigid cell with a higher MCHC value, we set *μ*_REF_ = (250–500) *μ*_0_. In addition, for the hypoxia-unaffected RBCs in each cell density fraction, we assume comparable deformability with the population in the same cell density fraction under the Oxy state. Similar parameters have been used as inputs for DPD simulations to quantify the shear-independent rheological behavior of blood flow in SCD [32]. We also investigated the functional dependence of shear viscosity of sickle RBC suspensions on the effective shear modulus, and demonstrated that the sickle RBC suspension behaves as a Newtonian fluid only if the effective shear modulus of sickle RBCs increases by two or three orders of magnitude [31]. The bending rigidity *k*_c_, following prior work [31,32], is kept constant in the model as the hemoglobin concentration increased.

The cell sickling delay time due to intracellular HbS polymerization is demonstrated to be the primary determinant of clinical severity in SCD [41]. The delay time of cell sickling is extraordinarily sensitive to solution conditions, particularly to HbS concentration. From prior results obtained from *in vitro* microfluidics experiment on sickle RBCs [23] and *in vivo* studies [41], we set the mean *t*_D_ ≈ 8.7 s, 19.8 s and 23.6 s for granular, elongated and classic sickle shaped RBCs, respectively, for the off-HU group blood samples. In the microfluidic experiments, the delay time for sickling for the on-HU group blood samples varied from 28 to 100 s [23], which is much longer than that for the off-HU group. Based on the experimental results, here we set the mean *t*_D_ to be in the same range for the on-HU group. The cell unsickling delay time, *t*_D,R_, is also directly obtained from the microfluidic experiments. According to Du *et al*. [23], the cell unsickling process after reoxygenation (ReOxy) was much faster (< 20 s) than the cell sickling process, and the delay time distribution of cell unsickling was not significantly different between the two different groups. Based on the experimental results, here we set the mean *t*_D,R_ to be in the range of 10–15 s.

All simulations were carried out with a representative volume of 180 μm × 60 μm × 5 μm that comprised a total of 30 periodic obstacles with a fluid particle number density of 3 obtained by dividing the fluid particle number of the model system by its volume. Capillary obstruction statistics were collected by running 200 independent simulations and the collective behavior is found to be independent of the initial position and initial orientation of RBCs. For the case of flow under Oxy state (without obstruction), steady state is reached with an average velocity of ~ 120 μm/s. The simulations are performed using the _*USER*_MESO functional package that was written based on LAMMPS [42]. The time integration of the motion equations is computed through a modified velocity–Verlet algorithm with λ = 0.5 and time step Δt = 0.001 τ where τ is a characteristic time in DPD units. A typical simulation performed in the current study involves one million time steps and a computing time, on average, of about 20,000 CPU core hours on the Blue Gene/P system at the Argonne Leadership Computing Facility (ALCF).

## Results/Discussion

### Behavior of individual sickle cells under transient hypoxia

**Fig 2:**
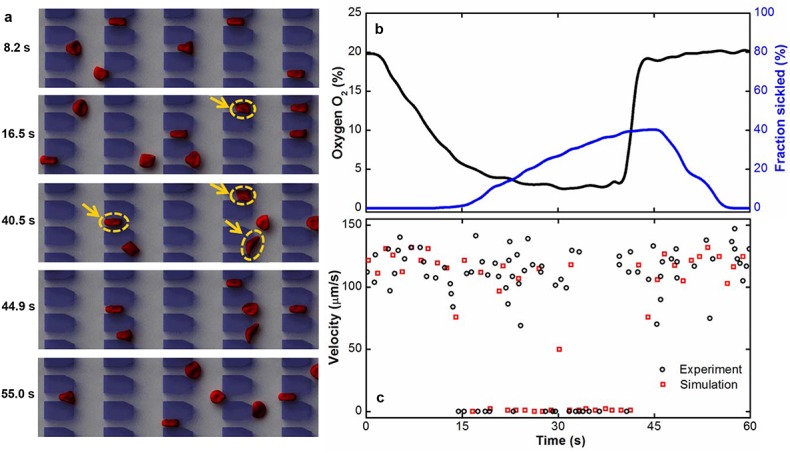
Dynamic behavior of individual sickle cells in response to changes in oxygen concentration. (a) Simulations of time sequence of RBCs flowing through capillary-inspired microchannel under transient hypoxia. The RBCs flow from right to left. Arrows indicate the trapped sickle cells at the microgates. (b) A representative profile of the sickled fraction under transient hypoxia in DPD simulation. In this case, the sickled fraction is changed from 0.0% to 20.2% by lowering the O_2_ concentration from 20% (Oxy state) to less than 5% (DeOxy state). (c) Representative velocity profiles of individual sickle cells obtained through experimental measurement (red circles) and numerical simulations (black squares).

Individual sickle cells show marked heterogeneity, and the variance is considerable even within the same density-fraction. This leads to the question: how do individual sickle RBCs from different density-fraction behave differently from healthy ones when they travel though microcapillaries under transient hypoxic conditions? Here, we investigate the motion of individual RBCs flowing in a capillary-inspired microchannel that consists of parallel periodic obstacles forming 15-μm-long, 4-μm-wide and 5-μm-high microgates. Fig 2 and S2 Video in supplementary material, show typical dynamic motion of RBCs travelling through the microfluidic channel under cyclic hypoxia. This figure and the accompanying video reveal that all RBCs are easily deformed and pass readily through the microgates. Following a decrease in O_2_ concentration, some RBCs become sickled and get trapped at the microgates; other RBCs can still deform and squeeze through the microgates (Fig 2). When hypoxia continues for a prolonged period, more RBCs become sickled and are obstructed at the microgates. After the O_2_ levels are restored, these rigid RBCs remain trapped for a few seconds due to a delay in the cell unsickling process before regaining their deformability; after this delay period, they once again easily traverse the microgates (Fig 2). The simulated individual cell behavior under controlled hypoxia conditions is in qualitative agreement with experimental observations [23]. In addition, we calculated the transit velocity of individual sickle RBCs under transient hypoxia and compared them to experimentally measured data, see Fig 2. We found that the transit velocities obtained from experiments and simulations are mutually consistent, under both Oxy and DeOxy states.

**Fig 3:**
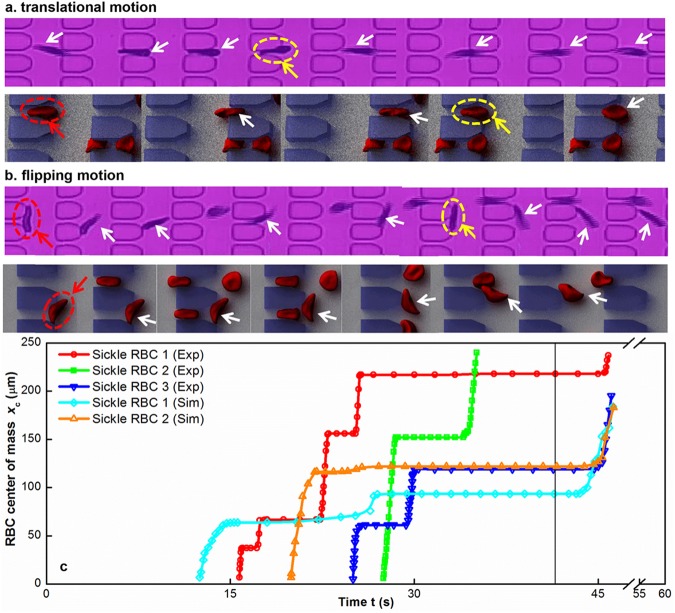
Direct observation of single-cell capillary obstruction. Four sequences of video images of hypoxia-affected sickle RBCs (indicated by white arrows) passing through microchannel constrictions before they are obstructed at the microgates. These sickle RBCs have translational (a) and flipping (b) motion, leading to parallel (a) or vertical (b) blockage, which may cause a transient (indicated by yellow arrows) or persistent (indicated by red arrows) blockage to blood flow. (c) Individual trajectories of hypoxia-affected sickle RBCs passing through microchannel constrictions. Each line shows an individual trajectory of a hypoxia-affected sickle RBC in microfluidic channel. The line segments, parallel to the horizontal axis, indicate transient or persistent (the topmost one, if any) occlusions caused by trapped sickle RBCs.

A direct means of triggering the early stages of a vaso-occlusive event is possible by following trajectories of sickle RBCs under controlled transient hypoxia at the single-cell level. For the purpose of illustration and to help with the analysis, we track the trajectories of individual sickle RBCs flowing in the capillary-inspired microchannel, both from patient-specific predictive simulations and companion microfluidic experiments, see Fig 3. These figures show that the sickle RBCs have two different types of motion, *i*.*e*., translational and flipping motion, before they arrest at the microgates. These different types of motion may lead to transient or even permanent occlusion of the microcapillary. We observe that a sickle RBC favors moving along the flow direction (translational motion) when it undergoes cell sickling (Fig 3 and corresponding S3–S5 Videos). Specifically, for elongated and crescent-shaped (classic sickle) cells, more than three quarters of the cells follow the translational motion. The flow of an RBC approaching the entrance of a microgate is disturbed because of the size mismatch between the cell and microgate. While a deformable cell is able to squeeze through the microgate, a stiffened (sickled) cell loses its ability to deform dynamically. Instead, it undergoes a continuous rotation until its long axis is nearly parallel to the flow, allowing a part of the cell to enter into the microgate. These cells are usually arrested at the microgates in a parallel manner, *i*.*e*., the sickle RBC tends to align with flow streamlines, as shown in Fig 3. Our observations show that such a blockage is initially transient (Fig 3; S3–S5 Videos), due to a relatively smaller contact area of the trapped sickle RBC than the cross-sectional area of the microgates. The trapped sickle RBC may move slowly through the microgates (S3 Video), or eventually stop at the microgates, causing persistent obstruction to RBC flow (S4 and S5 Videos), if the sickle RBCs become stiff, e.g., an ISC in fraction IV. Interestingly, if a sickle RBC moves alongside with other RBCs, while travelling or being trapped, it may also experience considerable rotation because it is subject to a velocity gradient even when the sickle RBC aligns with flow streamlines. Such a cell is more likely to flip, leading to occasionally longitudinal or vertical blockage (sickled RBC aligns perpendicular to flow direction) (Fig 3 and corresponding S3 Video and S6 Video). It should be noted that the vertical blockage is much more likely to become persistent blockage, with more serious implications for vaso-occlusion in SCD patients.

**Fig 4:**
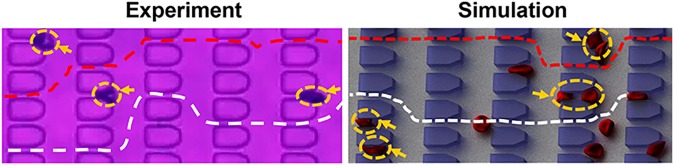
Trajectories of deformable sickle cells flowing in microfluidic channels. Each line shows the trajectory of individual deformable sickle RBCs in microfluidic channel flow. Arrows indicate the trapped sickle RBCs at the microgates.

We also track the dynamic behavior of individual sickle RBCs at different density-fractionated subpopulations under controlled transient hypoxic conditions. Deformable sickle RBCs in fractions I and II appear to take a preferred path (if the adjacent microgates in the flow direction are not fully blocked): they twist and turn along a *serpentine path* once they spot trapped cells ahead of them, see Fig 4. However, the stiff sickle RBCs in fractions III and IV just flow toward the trapped sickle RBCs and eventually stop nearby (S7 and S8 Videos). The difference in cell behavior between deformable and stiff sickle RBCs is probably due to the chaotic motion of RBCs caused by cell-cell interactions and flow perturbations near the obstructions. For a deformable sickle RBC, fluid flow can change the cell shape substantially and drift it along the flow direction; however, a stiff sickle RBC is also disturbed by the fluid flow near the obstruction.

### Morphological and mechanical factors involved in vaso-occlusive crisis

**Fig 5:**
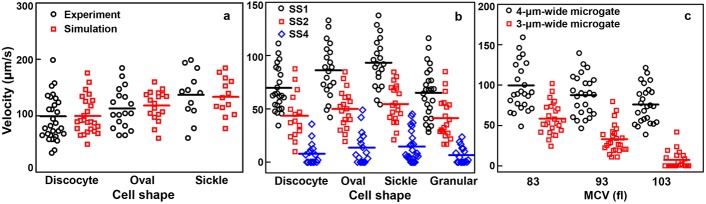
Dependence of cell biodynamics on sickle cell shapes and mean cell volume in the Oxy and DeOxy states. (a) Transit velocity of sickle cells with different shapes in the Oxy state obtained from experiment (black circles) and simulation (red squares). (b) Simulations of transit velocity of sickle cells in fractions I (SS1, black circles), II (SS2, red squares), and IV (SS4, blue diamonds) in the DeOxy state. (c) Simulations of transit velocity of individual sickle RBCs with different cell volume through 4-μm-wide (black circles) and 3-μm-wide (red squares) microgates.

SCD exhibits heterogeneous morphologies, which depend on the DeOxy rate [10]. Gradual DeOxy is known to result in predominantly elongated- and crescent-shaped RBCs, whereas rapid DeOxy results in less distorted granular-shaped RBCs. Cell morphological sickling is thus identified by visibly changes in cell shape and texture associated with transient hypoxic conditions. We performed computational simulations of patient-based samples with different cell shape count information under the Oxy and DeOxy states. We calculated the transit velocity of sickle RBCs in the microfluidic channel and compared the results to those measured in experiments (Fig 5). The results show that the RBC shape plays an important role in cell traversal through microgates: with the same cell rigidity values under the Oxy state, sickle RBCs with a disc-shape have the lowest transit velocity (*v* ~ 98 μm/s). By contrast, sickle RBCs with a crescent shape have the highest transit velocity (*v* ~ 136 μm/s). Therefore, the transit velocity of sickle cells with different shapes is statistically significantly different. The reason for this might stem from the different cross-sectional areas of sickle RBCs with different shapes. In our simulations, the cross-sectional area of sickle RBCs with disc, oval, and crescent shapes are about 32 μm^2^, 26 μm^2^, 23 μm^2^, respectively, which are greater than that of the microgate opening (20 μm^2^). Thus, the sickle RBC has to deform during its traversal through the microgates. When we applied a fixed pressure gradient in each simulation to force the cells to cross the microgate, the granular-shaped ones suffer the largest deformation, resulting in the lowest transit velocity. The simulation results, albeit counter-intuitive, are quantitatively consistent with the experimental observations of sickle RBCs transiting in the microfluidic channel.

It is known that the loss of deformability of RBCs play a crucial role in vaso-occlusion in SCD [11,14,40]. We perform simulations of sickle RBCs in different density-fractionated subpopulations in order to verify the significant role of cell deformability in determining the dynamic and rheological characteristics of individual sickle RBCs. Here, we consider three distinct types of sickle RBCs under channel flow, *i*.*e*., the hypoxia-affected RBCs in fractions I (SS1), II (SS2), and IV (SS4). Our simulations indicate that sickle RBCs show increased flow resistance under the DeOxy state, especially for the sickle RBCs in fraction IV; some sickle RBCs (for example, the granular and disc-shaped ones) in this fraction always are obstructed at the microgates (Fig 5). In addition, considering the sickle RBCs in the same density-fractioned subpopulation, (hence with nearly the same shear modulus), the present results also show an obvious difference in transit velocity for sickle RBCs with different shapes. This confirms the hypothesis that SCD exhibits substantial heterogeneity even within the same density-fractionated subpopulation.

There is currently no universal cure for SCD patients, but symptoms can be managed with fluids, oxygen and medication. HU has a number of characteristics of an ideal drug for SCD patients [43,44]. It increases HbF production and reduces the occurrence of sickling-related complications [45], partially attributed to improved cell hydration [46] and cell deformability [47,48]. However, HU treatment is also associated with an elevated MCV [49,50]. This dual influence of HU on RBC structural and mechanical properties may significantly affect cell traversal through the microgates. In order to develop a quantitative assessment of the efficacy of HU treatment on the dynamic behavior of individual sickle RBCs, we examine how the enlarged sickle RBCs move in shear flow.

We study the dynamic behavior of individual sickle RBCs using patient-specific hematological values for individual patients with SCD after treated with HU. When the MCV values are compared between the off-HU and on-HU groups, we find that they are always higher in the on-HU groups (see the MCV values in Table 1), consistent with previous observations [49,50]. Our simulations indicate that sickle RBCs travelling through the microgates are sensitive to MCV, and that the correlation between cell transit velocity and MCV is enhanced with the decrease in the size of the microgates. As shown in Fig 5, an increase in MCV from 83 fl to 103 fl results in a slight decrease in cell transit velocity when individual sickle RBCs travel through 4-μm-wide microgates. The average cell transient velocity decreases significantly, however, when the individual sickle RBCs pass through 3-μm-wide microgates. These results reveal the importance of MCV as a determinant of individual sickle RBCs passage through the smallest capillary.

### Single-cell capillary obstruction

The hypoxia-affected RBCs in SCD cause blockages at the microgates under the DeOxy state. Hence, we performed simulations of patient-based samples to predict the single-cell capillary obstruction and compare the results against experimental data [23].

**Fig 6:**
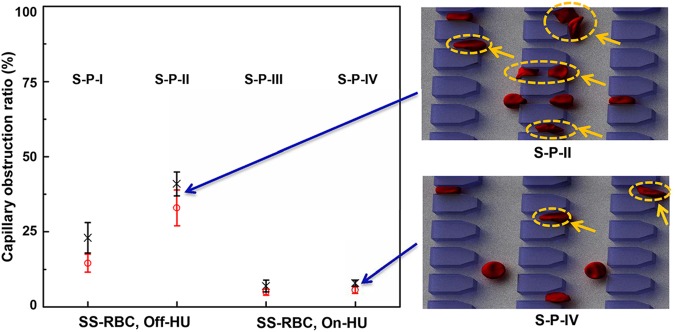
Microfluidic studies of single-cell capillary obstruction for blood samples of on-HU groups and off-HU groups. The cross symbols show the measured experimental data and the circles show the simulation results.

The capillary obstruction ratio, is defined as the ratio of the total number of trapped sickle RBCs at the microgates to the total number of RBCs in the microfluidic channel during the DeOxy state. This ratio is now examined from the four SCD blood samples including on-HU and off-HU groups. Fig 6 shows the values of capillary obstruction ratio obtained from DPD simulations and experiments. In general, the off-HU group exhibits a significantly higher capillary obstruction ratio than the on-HU group. Such increases are likely caused primarily by changes in sickled fraction. As shown in Table A in S1 Text, 20.2% and 39.0% of RBCs sickle in the off-HU/S-P-I group and off-HU/S-P-II group, respectively, among which 15.8% and 39.0% of hypoxia-affected RBCs fall within cell density fractions III and IV, respectively. In the on-HU groups, fewer RBCs become sickled than those in the on-HU groups, *i*.*e*., there are only 9.4% and 3.1% of sickled RBCs in the on-HU/S-P-III group and the on-HU/S-P-IV group, respectively, among which only 6.3% and 3.1% of hypoxia-affected RBCs fall within cell density fractions III and IV, respectively. As we demonstrated in an earlier section, the sickled RBCs show increased flow resistance under the DeOxy state and the densest ones are usually unable to traverse the microgates. Thus, a relatively large number of sickle RBCs, especially in the denser cell fractions, can cause a marked increase in single-cell capillary obstruction. The results confirm that cell deformability plays a key role in RBC traversal in small microfluidic channels. In addition, we find that the predictions of all DPD simulation cases fall within the range of experimental data. The discrepancy between predictions and experiments could arise from an underestimation of the cell morphological sickling count of individual sickle RBCs.

In summary, in this paper, we demonstrate the unique capabilities benefits of combining dynamic microfluidic experiments with multiscale simulations for characterizing the complex behavior of individual sickle RBCs in a capillary-like microenvironment under transient hypoxia. We show that hypoxia-affected RBCs undergoing sickling significantly alter cell behavior. We also monitor the dynamic behavior of both hypoxia-affected and hypoxia-unaffected RBCs as they travel through the capillary-like microenvironment under cyclic hypoxia. Taken together, these experiments and corresponding systematic particle-based simulations elucidate the effects of irregular geometry, decreased cell deformability, and elevated cell volume on the biodynamic behavior of individual sickle RBCs and their roles in single-cell capillary obstruction. They provide a quantitative measure of the heterogeneity associated with SCD at the single-cell level. Hence, the particle-based simulations and comparisons with available independent experiments offer a powerful means for real-time monitoring of *in vitro* behavior of individual sickle RBCs under controlled transient hypoxic conditions, providing an objective way of assessing the effectiveness of targeted drug therapy aimed at easing or preventing vaso-occlusive crisis associated with SCD.

Our model does not explicitly include the intracellular HbS polymer fibers. Hence, it does not model the interaction between cell membrane and polymer fibers, and the potential influences on morphological distortion of RBCs and the attendant alteration in their mechanical properties. This deficiency could be addressed in future work by recourse to a hybrid model that encompasses the molecular and cellular scales by combing the MS-RBC model with particle-based HbS polymer models developed recently [51–53]. In addition, it would require further computational validation and extensive testing of these patient-specific models against clinical and experimental studies to make future predictions more reliable. Such simulations from these patient-specific predictive models would be useful for testing known biomarkers and discovering new biomarkers.

## Supporting information

S1 Text Supplementary material. (DOC)

S1 Video Simulation of RBC morphological sickling and unsickling process under controlled transient hypoxic conditions in shear flow. (MP4)

S2 Video Simulation of sickle RBCs flowing in capillary-like microenvironment under transient hypoxic conditions. The hypoxia-affected RBCs become stiffer than other RBCs, so they tend to get stuck at the microgates, and block the blood flow.
(MP4)

S3 Video An individual sickle RBC (Sickle 1, exp; red arrow) travels through the microgates in a translational motion, causing a transient occlusion before moving out the capillary-like microenvironment. The movie also shows that another individual sickle RBC (Sickle 3, exp; yellow arrow) travels through the microgates in a flipping motion, leading to a persistent occlusion.
(MP4)

S4 Video Individual sickle RBC (Sickle 2, exp) travels through the microgates in a translational motion, causing sequential transient occlusion before a completely blockage at the microgates (persistent occlusion). (MP4)

S5 Video Simulation of individual sickle RBC (Sickle 1, sim) traveling through the microgates in a translational motion, causing a transient occlusion before a completely blockage at the microgates. (MP4)

S6 Video Simulation of individual sickle RBC (Sickle 2, sim) traveling through the microgates in a flipping motion, causing a rapidly persistent occlusion. (MP4)

S7 Video A stiff sickle RBC flows through blockages. It just moves toward the blockage and get stuck there.
(MP4)

S8 Video Simulation of stiff sickle RBC moving toward one trapped sickle RBC and eventually stopped nearby. (MP4)

## References

[1] Schubert C. Single-cell analysis: The deepest differences. Nature. 2011; 480: 133–137. 10.1038/480133a 22129730

[2] Modell B, Darlison M. Epidemiology of haemoglobin disorders and derived service indicators. Bull World Health Organ. 2008; 86: 480–487. 10.2471/BLT.06.036673 18568278 PMC2647473

[3] Gravitz L, Pincock S. Sickle-cell disease. Nature. 2014; 515: S1 10.1038/515S1a 25390134

[4] Pauling L, Itano HA, Singer SJ, Wells IC. Sickle cell anemia, a molecular disease. Science. 1949; 110: 543–548. 10.1126/science.110.2865.543 15395398

[5] Samuel RE, Briehl RW. Nucleation and growth of fibres and gel formation in sickle cell haemoglobin. Nature. 1990; 345: 833–835. 10.1038/345833a0 2359460

[6] Liu SC, Derick LH, Zhai S, Palek J. Uncoupling of the spectrin-based skeleton from the lipid bilayer in sickled red cells. Science. 1991; 252: 574–576. 10.1126/science.2020854 2020854

[7] Kaul DK, Fabry ME, Windisch P, Baez S, Nagel RL. Erythrocytes in sickle cell anemia are heterogeneous in their rheological and hemodynamic characteristics. J Clin Invest. 1983; 72: 22–31. 10.1172/JCI110960 6874947 PMC1129157

[8] Christoph GW, Hofrichter J, Eaton WA. Understanding the shape of sickled red cells. Biophys J. 2005; 88: 1371–1376. 10.1529/biophysj.104.051250 15542552 PMC1305139

[9] Fabry ME, Mears JG, Patel P, Schaeferrego K, Carmichael LD, et al Dense cells in sickle-cell-anemia—the effects of gene interaction. Blood. 1984; 64: 1042–1046. 6207871

[10] Kaul DK, Xue H. Rate of deoxygenation and rheologic behavior of blood in sickle cell anemia. Blood. 1991; 77: 1353–1361. 2001458

[11] Itoh T, Chien S, Usami S. Effects of hemoglobin concentration on deformability of individual sickle cells after deoxygenation. Blood. 1995; 85: 2245–2253. 7718897

[12] Higgins JM, Eddington DT, Bhatia SN, Mahadevan L. Sickle cell vasoocclusion and rescue in a microfluidic device. Proc Natl Acad Sci USA. 2007; 104: 20496–20500. 10.1073/pnas.0707122105 18077341 PMC2154459

[13] Hosseini P, Abidi SZ, Du E, Papageorgiou DP, Choi Y, Park Y, Higgins JM, Kato GJ, Suresh S, Dao M, Yaqoob Z, So PT. Cellular normoxic biophysical markers of hydroxyurea treatment in sickle cell disease. Proc Natl Acad Sci USA. 2016; 113: 9527–9532. 10.1073/pnas.1610435113 27512047 PMC5003247

[14] Chien S, Usami S, Bertles JF. Abnormal rheology of oxygenated blood in sickle cell anemia. J Clin Invest. 1970; 49: 623–634. 10.1172/JCI106273 5443167 PMC322516

[15] Barabino GA, Platt MO, Kaul DK. Sickle cell biomechanics. Annu Rev Biomed Eng. 2010; 12: 345–367. 10.1146/annurev-bioeng-070909-105339 20455701

[16] Wood DK, Soriano A, Mahadevan L, Higgins JM, Bhatia SN. A biophysical indicator of vaso-occlusive risk in sickle cell disease. Sci Transl Med. 2012; 4: 123ra26 10.1126/scitranslmed.3002738 22378926 PMC3633235

[17] Lei H, Karniadakis GE. Probing vasoocclusion phenomena in sickle cell anemia via mesoscopic simulations. Proc Natl Acad Sci USA. 2013; 110: 11326–11330. 10.1073/pnas.1221297110 23798393 PMC3710812

[18] Mirnezami R, Nicholson J, Darzi A. Preparing for Precision Medicine. N Engl J Med. 2012; 366: 489–491. 10.1056/NEJMp1114866 22256780

[19] Collins FS, Varmus H. A New Initiative on Precision Medicine. N Engl J Med. 2015; 372: 793–795. 10.1056/NEJMp1500523 25635347 PMC5101938

[20] Kohane IS. Ten things we have to do to achieve precision medicine. Science. 2015; 349: 37–38. 10.1126/science.aab1328 26138968

[21] Seoane J, Mattos-Arruda LD. The challenge of intratumour heterogeneity in precision medicine. J Intern Med. 2014; 276: 41–51. 10.1111/joim.12240 24661605

[22] Li X, Li H, Chang HY, Lykotrafitis G, Karniadakis GE. Computational biomechanics of human red blood cells in hematological disorders. J Biomech Eng. 2017; 139: 020804 10.1115/1.4035120 27814430 PMC5395917

[23] Du E, Diez-Silva M, Kato GJ, Dao M, Suresh S. Kinetics of sickle cell biorheology and implications for painful vasoocclusive crisis. Proc Natl Acad Sci USA. 2015; 112: 1422–1427. 10.1073/pnas.1424111112 25605910 PMC4321273

[24] Pivkin IV, Karniadakis GE. Controlling density fluctuations in wall-bounded dissipative particle dynamics systems. Phys Rev Lett. 2006; 96: 206001 10.1103/PhysRevLett.96.206001 16803187

[25] Pivkin IV, Karniadakis GE. Accurate coarse-grained modeling of red blood cells. Phys Rev Lett. 2008; 101: 118105 10.1103/PhysRevLett.101.118105 18851338

[26] Fedosov DA, Caswell B, Karniadakis GE. A multiscale red blood cell model with accurate mechanics, rheology, and dynamics. Biophys J. 2010; 98: 2215–2225. 10.1016/j.bpj.2010.02.002 20483330 PMC2872218

[27] Fedosov DA, Pan WX, Caswell B, Gompper G, Karniadakis GE. Predicting human blood viscosity in silico. Proc Natl Acad Sci USA. 2011; 108: 11772–11776. 10.1073/pnas.1101210108 21730178 PMC3141939

[28] Turlier H, Fedosov DA, Audoly B, Auth T, Gov NS, Sykes C, et al Equilibrium physics breakdown reveals the active nature of red blood cell flickering. Nature Phys. 2016; 12: 513–519. 10.1038/nphys3621

[29] Fedosov DA, Caswell B, Suresh S, Karniadakis GE. Quantifying the biophysical characteristics of Plasmodium-falciparum-parasitized red blood cells in microcirculation. Proc Natl Acad Sci USA. 2011; 108: 35–39. 10.1073/pnas.1009492108 21173269 PMC3017137

[30] Pivkin IV, Peng Z, Karniadakis GE, Buffet PA, Dao M, Suresh S. Biomechanics of red blood cells in human spleen and consequences for physiology and disease. Proc Natl Acad Sci USA. 2016; 113: 7804–7809. 10.1073/pnas.1606751113 27354532 PMC4948333

[31] Li XJ, Du E, Lei H, Tang YH, Dao M, Suresh S, et al Patient-specific blood rheology in sickle-cell anemia. Interface Focus. 2016; 6: 20150065 10.1098/rsfs.2015.0065 26855752 PMC4686241

[32] Lei H, Karniadakis GE. Quantifying the rheological and hemodynamic characteristics of sickle cell anemia. Biophys J. 2012; 102: 185–194. 10.1016/j.bpj.2011.12.006 22339854 PMC3260690

[33] Le Floch-Yin FT. Design of a numerical model for simulation of blood microcirculation and study of sickle cell disease [Ph.D. thesis]. Massachusetts Institute of Technology, USA; 2010, 69–74.

[34] Chien S, King RG, Kaperonis AA, Usami S. Viscoelastic properties of sickle cells and hemoglobin. Blood Cells. 1982; 8: 53–64. 7115978

[35] Evans E, Mohandas N, Leung A. Static and dynamics rigidities of normal and sickle erythrocytes: Major influence of cell hemoglobin concentration. J Clin Invest. 1984; 73: 477–488. 10.1172/JCI111234 6699172 PMC425039

[36] Evans EA, Mohandas N. Membrane-associated sickle hemoglobin: a major determinant of sickle erythrocyte rigidity. Blood. 1987; 70: 1443–1449. 3663941

[37] Ballas SK, Mohandas N. Sickle red cell microrheology and sickle blood rheology. Microcirculation. 2004; 11: 209–225. 10.1080/10739680490279410 15280093

[38] Klug PP, Lessin LS, Radice P. Rheological aspects of sickle cell disease. Arch. Intern. Med. 1974; 133: 577–590. 10.1001/archinte.1974.00320160071007 4594395

[39] Dong C, Chadwick RS, Schechter AN. Influence of sickle hemoglobin polymerization and membrane properties on deformability of sickle erythrocytes in the microcirculation. Biophys. J. 1992; 63: 774–783. 10.1016/S0006-3495(92)81655-7 1420913 PMC1262210

[40] Itoh T, Chien S, Usami S. Deformability measurements on individual sickle cells using a new system with P_O2_, and temperature control. Blood. 1992; 79: 2141–2147. 1562740

[41] Mozzarelli A, Hofrichter J, Eaton W. Delay time of hemoglobin S polymerization prevents most cells from sickling in vivo. Science. 1987; 237: 500–506. 10.1126/science.3603036 3603036

[42] Tang Y-H, Karniadakis GE. Accelerating dissipative particle dynamics simulations on GPUs: Algorithms, numerics and applications. Comput. Phys. Comm. 2014; 185: 2809–2822. 10.1016/j.cpc.2014.06.015

[43] Charache S, Dover GJ, Moore RD, Eckert S, Ballas SK, Koshy M, et al Hydroxyurea: effects on hemoglobin F production in patients with sickle cell anemia. Blood. 1992; 79: 2555–2565. 1375104

[44] Ferster A, Tahriri P, Vermylen C, Sturbois G, Corazza F, Fondu P, et al Five years of experience with hydroxyurea in children and young adults with sickle cell disease. Blood. 2001; 97: 3628–3632. 10.1182/blood.V97.11.3628 11369660

[45] Akinsheye I, Alsultan A, Solovieff N, Ngo D, Baldwin C, Sebastiani P, et al Fetal hemoglobin in sickle cell anemia. Blood. 2011; 118: 19–27. 10.1182/blood-2011-03-325258 21490337 PMC3139383

[46] Ballas SK, Dover GJ, Charache S. Effect of hydroxyurea on the rheological properties of sickle erythrocytes in vivo. Am J Hematol. 1989; 32: 104–111. 10.1002/ajh.2830320206 2757007

[47] Brando MM, Fontes A, Barjas-Castro ML, Barbosa LC, Costa FF, Cesar CL, et al Optical tweezers for measuring red blood cell elasticity: application to the study of drug response in sickle cell disease. Eur J Haematol. 2003; 70: 207–211. 10.1034/j.1600-0609.2003.00027.x 12656742

[48] Byun H, Hillman TR, Higgins JM, Diez-Silva M, Peng Z, Dao M, et al Optical measurement of biomechanical properties of individual erythrocytes from a sickle cell patient. Acta Biomater. 2012; 8: 4130–4138. 10.1016/j.actbio.2012.07.011 22820310 PMC3576574

[49] Fattori A, de Souza RA, Saad ST, Costa FF. Acute myocardial infarction in sickle cell disease: a possible complication of hydroxyurea treatment. Hematol J. 2005; 5: 589–590. 10.1038/sj.thj.6200572 15692605

[50] Ware R. How I use hydroxyurea to treat young patients with sickle cell anemia. Blood. 2010; 115: 5300–5311. 10.1182/blood-2009-04-146852 20223921 PMC2902131

[51] Li XJ, Caswell B, Karniadakis GE. Effect of chain chirality on the self-assembly of sickle hemoglobin. Biophys. J. 2012; 103: 1130–1140. 10.1016/j.bpj.2012.08.017 22995485 PMC3446663

[52] Lu L, Li XJ, Vekilov PG, Karniadakis GE. Probing the twisted structure of sickle hemoglobin fibers via particle simulations. Biophys J. 2016; 110: 2085–2093. 10.1016/j.bpj.2016.04.002 27166816 PMC4940994

[53] Li X, Dao M, Lykotrafitis G, Karniadakis GE. Biomechanics and biorheology of red blood cells in sickle cell anemia. J Biomech. 2017; 50: 34–41. 10.1016/j.jbiomech.2016.11.022 27876368 PMC5368081

