# Supplementary material for: Patient-specific modeling of individual sickle cell behavior under transient hypoxia
Source: PLoS Comput Biol. 2017 Mar 13;13(3):e1005426. doi: 10.1371/journal.pcbi.1005426 (PMC5367819; doi:10.1371/journal.pcbi.1005426)
Supplement: S1 Text — (DOC) [file pcbi.1005426.s001.doc]

**Supporting Information:**

**Patient-specific modeling of individual sickle cell behavior under transient hypoxia**

**Xuejin Li, E Du, Ming Dao, Subra Suresh, and George Em Karniadakis**

**Dissipative particle dynamics method**

Dissipative Particle Dynamics (DPD) [1, 2] is a particle-based mesoscopic simulation technique that allows modeling of fluids and soft matter. In a DPD simulation, a particle represents the center of mass in a cluster of atoms, and the position and momentum of the particle is updated in a continuous phase but spaced at discrete time steps. Particles *i* and *j* at positions *r_i_* and *r_j_* interact with each other via pairwise conservative, dissipative, and random forces, which are given by:


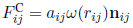
 (1)


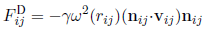
 (2)


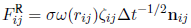
 (3)

where **r***_ij_* = **r***_i_* - **r***_j_*, *r_ij_* = |**r***_ij_*|, **n***_ij_* = **r***_ij_* / *r_ij_*, and **v***_ij_* = **v***_i_* - **v***_j_*. The coefficients *a_ij_* , γ and σ define, respectively, the strength of conservative, dissipative and random forces. The last two coefficients are coupled with the temperature of the system by the fluctuation-dissipation theorem as σ^2^ = 2γ*k*_B_T. In addition, ζ*_ij_* is a random number with zero mean and unit variance. The weight function *ω*(*r_ij_*) is given by


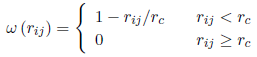
 (4)

where *r_c_* is the cutoff radius, which gives the extent of the interaction range.

The time integration of the motion equations is computed through a modified velocity-Verlet algorithm with time step Δ*t* = 0.001τ. It takes 2.0 ×10^7^ time steps for a typical simulation performed in the current study.

**Morphologic analysis of sickle RBCs**

Cell sickling was identified visually by changes in cell shape and cell texture associated with DeOxy. The morphology of sickled and unsickled cells was categorized into four major groups, including discoid- (D), granular- (G), elongated- (E) and crescent-shaped (C) RBCs. Selected characteristics and morphologic analysis of SCD patients are summarized in Table A.

**Table A. General characteristics and morphologic analysis of sickle RBCs in four representative SCD patients.**

**
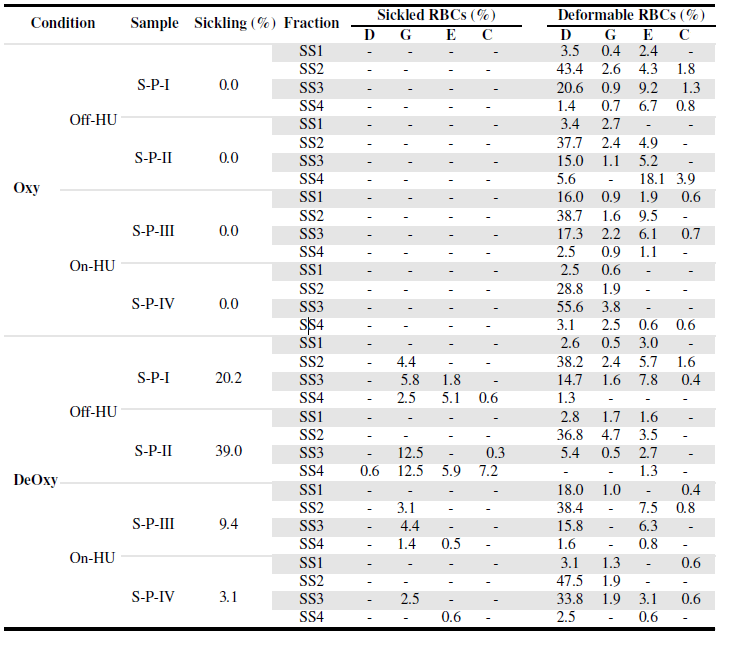
**Symbols D, G, E and C indicate discoid, granular, elongated and crescent (classic sickle) shapes.

**Table B. Stretching force exerted on the anchor points for three distinct types of sickle cell** morphology.

|  | **A** | **B** | **C** | **D** |
| --- | --- | --- | --- | --- |
| **Granular** | (0, 23, 31) | (-23, 0, 31) | (0, -23, 31) | (23, 0, 31) |
| **Elongated** | (0, 55, 11) | (0, 0, 0) | (0, -55, -11) | (0, 0, 0) |
| **Crescent** | (0, 55, 54) | (0, 0, 0) | (0, -55, 54) | (0, 0, 0) |

Unit: pN.

**Cell sickling profiles upon changes in O_2_ concentration**

Sickle RBCs show repeated sickling and unsickling in response to a cyclic hypoxia. However, the sickled fraction profiles are very much heterogeneous between different SCD patients. Representative cell sickling profiles upon changes in O_2_ concentration are shown in Figure A, which are used as input to the patient-specific computational simulations.


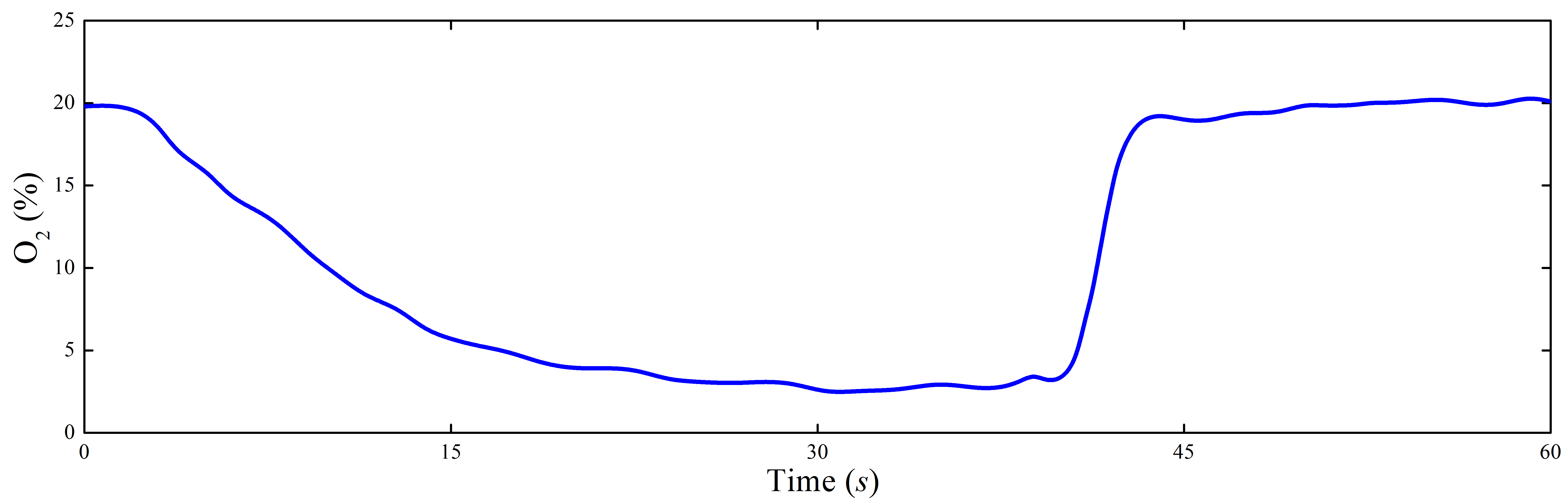


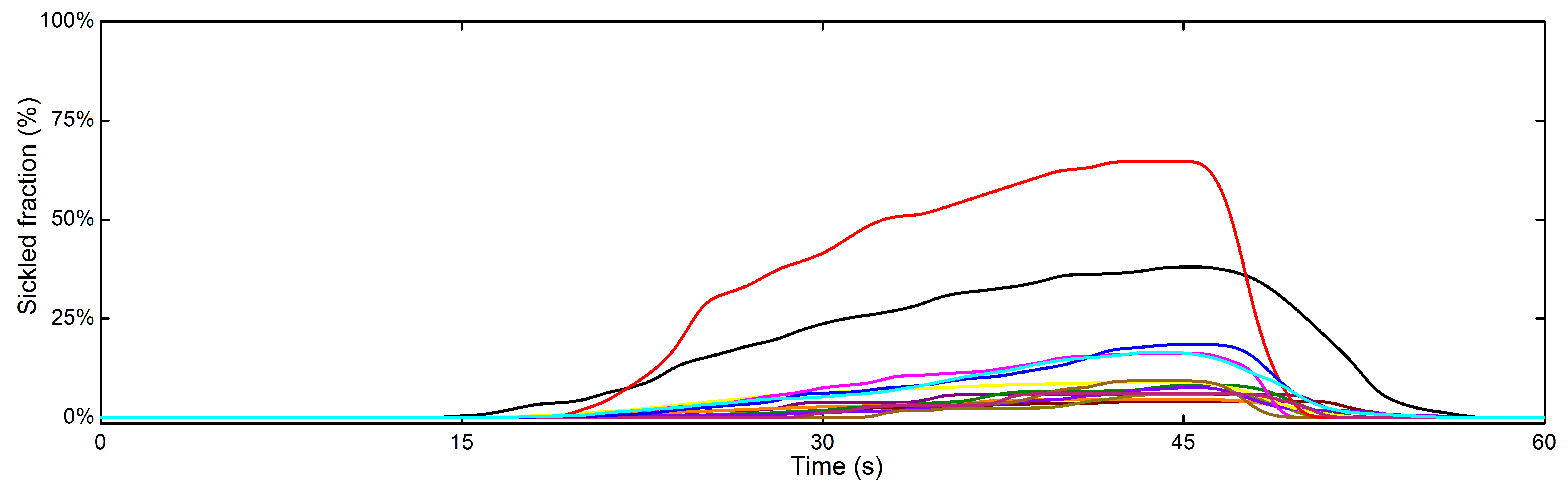


**Figure A. Time-dependence of O_2_ concentration (*Upper*) and sickled fraction profiles of multiple SCD samples during transient DeOxy (*Lower*).** Each curve represents an individual blood sample.

**Scaling of model and physical units**

In a DPD approach, it is convenient to use reduced units [2]. The unit of length is defined by the cutoff radius *r_c_*; the unit of the mass is defined by the mass of a particle; and the unit of energy is defined by *k*_B_T. It is difficult to have a precise idea of the scales involved in DPD simulations. The real size of a DPD particle may vary from one to several dozens of atoms, depending on the interaction potential and the time scale. A mapping strategy developed by Fedosov *et al.* [4] and then revised by Peng *et al.* [5] is adopted to provide an estimate of the physical length-, and time-scales in the DPD simulations of RBCs flow. Following their mapping strategy, we obtain the DPD length and time scale,


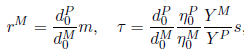
, (5)

where the superscript *M* denotes a quantity in reduced DPD units, while *P* identifies physical units. *d*_0_ is the cell diameter, *η*_0_ is a characteristic viscosity of fluid or RBC membrane, and *Y*_0_ is the membrane Young’s modulus. In the current simulations, the RBC diameter, the membrane Young’s modulus and the interior fluid viscosity are *d*_0_*^M^* = 7.8 *r^M^*, *Y*_0_*^M^* = 392.5 (*k*_B_T)*^M^*/(*r^M^*)^2^ and *η*_0_*^M^* = 18.8 (*k*_B_T)*^M^τ*/(*r^M^*)^3^, respectively, corresponding to *d*_0_*^P^* = 8.0 μm, *Y*_0_*^P^* = 18.9 μN/m, and *η*_i_ = 0.006 pN∙μm^-2^s (6 cP) in physical units, thus, the DPD length scale is *r^M^* ≈ 1.0 μm and time scale *τ* ≈ 6.8 ms.

**References**

1. P. J. Hoogerbrugge and J. M. V. A. Koelman. Simulating microscopic hydrodynamic phenomena with dissipative particle dynamics. Europhys. Lett., 19: 155-160, 1992.

2. R. D. Groot and P. B. Warren. Dissipative particle dynamics: Bridging the gap between atomistic and mesoscopic simulation. J. Chem. Phys., 107: 4423-4435, 1997.

3. E. A. Evans and R. Skalak. Mechanics and thermodynamics of biomembranes. CRC Press, Inc., Boca Raton, Florida, 1980.

4. D. A. Fedosov, B. Caswell, and G. E. Karniadakis. A multiscale red blood cell model with accurate mechanics, rheology, and dynamics. Biophys. J., 98: 2215-2225, 2010.

5. Z. L. Peng, X. J. Li, I. V. Pivkin, M. Dao, G. E. Karniadakis, and S. Suresh. Lipid-bilayer and cytoskeletal interactions in a red blood cell. Proc. Natl. Acad. Sci. U.S.A., 110: 13356-13361, 2013.
